# Renal metastasis of esophageal squamous cell carcinoma coexisting with adenoid cystic carcinoma: a case report and literature review

**DOI:** 10.3389/fonc.2026.1727349

**Published:** 2026-02-12

**Authors:** An-Qi Wu, Qiu-Hong Bao, Jian-Ming Pang, Qing-Qing Chen

**Affiliations:** Department of Oncology Medicine, Tiantai People’s Hospital of Zhejiang Province, Tiantai, Zhejiang, China

**Keywords:** adenocarcinoma, esophageal cancer, kidney metastasis, nephrectomy, squamous cell carcinoma

## Abstract

Esophageal cancer is one of the common malignancies worldwide. It frequently metastasizes to the lungs and liver, as well as to distant sites such as bones, heart, and brain. Clinically, renal metastasis is rare, but it is more frequently observed in autopsy specimens. The present case report describes a rare case of renal metastasis from esophageal squamous cell carcinoma coexisting with adenoid cystic carcinoma.

## Introduction

Esophageal cancer is an aggressive malignancy with high incidence and mortality rates. According to the World Health Statistics, esophageal cancer ranked 10th in global incidence and 6th in mortality in 2020 (1). Esophageal squamous cell carcinoma mainly occurs in the upper two-thirds of the esophagus, while adenocarcinoma predominantly arises in the lower one-third. In China and other East Asian countries, esophageal squamous cell carcinoma (ESCC) accounts for approximately 90% of esophageal cancer cases. Despite the availability of various treatment options, the prognosis remains bleak. Patients with advanced esophageal cancer typically have a median overall survival (OS) time of less than 1 year, with a 5-year survival rate of less than 16% (2). Although 50% to 70% of patients have the opportunity to receive curative resection, approximately half of these patients will still experience local recurrence or distant metastasis. The most common metastatic sites are the liver, lungs, bones, and adrenal glands.

Renal metastasis from esophageal cancer is rare, with only 20 cases reported previously. Herein, we present a case of a patient with esophageal cancer who developed renal metastasis more than 10 years after radical surgery, aiming to share experience in the diagnosis and management of this condition.

## Case presentation

**Figure 1:**
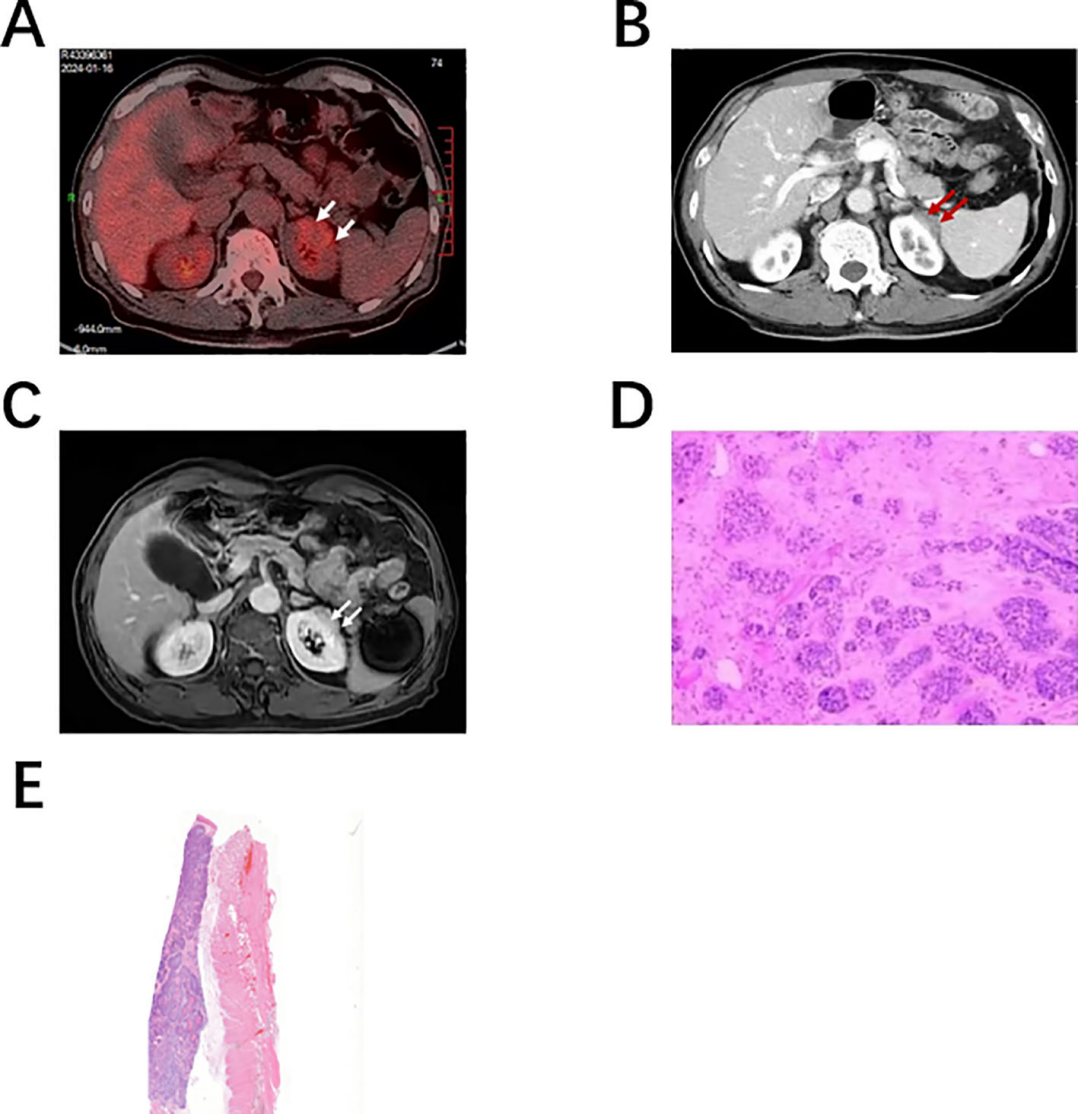
Patient’s imaging and pathological findings: **(A)** On January 14, 2024, PET-CT imaging revealed a soft tissue shadow at the outer margin of the upper pole of the left kidney, with heterogeneous elevation of FDG metabolism. Delayed scanning showed a slight increase in FDG metabolism. **(B)** On January 12, 2024, Enhanced abdominal CT scan showed an arcuate low-density shadow at the edge of the left kidney, approximately 28mm x 12mm in size. **(C)** On January 13, 2024, Enhanced MRI scan of the kidneys revealed an oval abnormal signal shadow in the left kidney, approximately 11 x 23mm in size. T1WI is iso-hypointense, T2WI is hyperintense, and DWI is hyperintense, and the lesion is progressively enhanced after enhancement. The perirenal fat space is clear and complete. The appearance of both adrenal glands is normal. No abnormally enlarged lymph nodes were seen in the retroperitoneum. **(D)** On January 26, 2024, Histological examination revealed squamous cell carcinoma with adenoid cystic carcinoma (H-E staining, ×200). **(E)** On October 8, 2014, Histological examination revealed squamous cell carcinoma with adenoid cystic carcinoma (H-E staining, ×100). The arrow indicates the location of the lesion.

On September 1, 2014, a 71-year-old male patient with a history of gallbladder stones and prostatic hyperplasia presented to our hospital due to a sensation of choking when eating. Pathological examination of gastroscopy biopsy specimens showed adenocarcinoma in the submucosa of the esophagus squamous epithelium, with a possibility of neuroendocrine tumor. On September 22, 2014, the patient underwent radical thoracoabdominal esophagectomy. On October 8, 2014, Postoperative pathology confirmed a diagnosis of ESCC with adenoid cystic carcinoma features (See **Figure 1E**). The tumor size was 7×4.5×2 cm, infiltrating the submucosa. No cancer involvement was found at either gastric or esophageal resection margins. No metastasis was found in the lymph nodes, including those adjacent to the recurrent laryngeal nerve, subcarinal, cardiac, middle and lower esophageal, and gastric. Immunohistochemical staining results: CK5/6 (+), p40 (+), p63 (+), CK7 (−), CK20 (−), CK19 (+), CD117 (+), Calponin (myoepithelial cells +), p63 (myoepithelial cells +), EGFR (focal weak +), Ki−67 (approximately 20%+), Pan−CK (+), Vimentin (−). (**Figure 1**) No adjuvant radiotherapy or chemotherapy was administered. Regular follow-up was performed.

On January 12, 2024, The patient does not have any discomfort, during a routine follow-up examination, Check tumor markers CEA 5.34 ng/ml、CA125 24.07U/ml、AFP 2.78 ng/ml, an abdominal enhanced computed tomography (CT) scan revealed (See **Figure 1B**) a space-occupying lesion located at the superior margin of the left kidney. Compared with the previous scan obtained on April 25, 2021, this lesion was newly identified. It was suspected to be neoplastic, with a possibility of renal lymphoma. On January 13, 2024, Renal enhanced magnetic resonance imaging (MRI) revealed (See **Figure 1C**) a malignant tumor in the left kidney, possibly papillary carcinoma or lymphoma. The possibility of metastatic tumor could not be excluded. On January 14, 2024, The whole-body PET-CT scan revealed(See **Figure 1A**) a soft tissue shadow with slightly low to equal density located at the outer margin of the upper pole of the left kidney. This shadow exhibited unevenly increased FDG metabolism, with a SUV value of 4.3. Delayed scanning further demonstrated a slight increase in FDG metabolism, with a SUV value of 5.3. On January 18, 2024, the patient underwent a successful robot-assisted laparoscopic partial nephrectomy. On January 26, 2024, Based on the postoperative pathology report(See **Figure 1D**), patient’s medical history, morphological findings, and immunohistochemistry, the left renal mass was diagnosed as a malignant tumor, considered a metastasis of esophageal basaloid squamous cell carcinoma with partial adenoid cystic carcinoma features. Both vascular and neural invasion were negative. Immunohistochemical staining results showed A2-1: P63(+), P40(+), Ki67(+, about 80% in hotspots), CK20(-), CK7(-), Vimentin (-), CD10(focal +), CAIX (-), CK5/6(focal +), Galectin-3(-), RCC (-), CD117(+), GATA3(-). (**Figure 1**) Comparing the histological morphology, immunohistochemistry, molecular characteristics, biological behavior, etc. in the patient’s postoperative pathology in 2014 and 2024, the patient was diagnosed with esophageal squamous cell carcinoma with renal metastasis, four cycles of adjuvant chemotherapy with carboplatin combined with paclitaxel were administered postoperatively, and regular follow-up was conducted: Years 1–2 after surgery (high-risk monitoring period): Tests every 3 months, including complete blood count (CBC), liver and kidney function tests, tumor markers (SCC, CEA, CYFRA21-1), contrast-enhanced thoracoabdominal CT scan (including full renal imaging), and esophagogastroduodenoscopy (EGD).Years 3–5 after surgery (medium-risk monitoring period): Tests every 6 months, including CBC, liver and kidney function tests, tumor markers, and contrast-enhanced thoracoabdominal CT scan (including full renal imaging); EGD once every 12 months. More than 5 years after surgery (low-risk follow-up period): Tests once a year, including tumor markers, liver and kidney function tests, and contrast-enhanced thoracoabdominal CT scan (including full renal imaging); EGD once every 2 years. To date, the patient is in good condition and continues to receive standardized follow-up.

## Discussion

The incidence of distant metastasis after radical resection of esophageal cancer ranges from 20% to 30%, with abdominal lymph nodes being the most common site of metastasis, followed by the liver, lungs, and bone (3). Metastasis to the kidney from esophageal cancer comprises only 4.8% of secondary renal tumors (4), which is significantly lower than the 12-13% autopsy rate reported in previous literature (5, 6). Clinically, metastasis of esophageal cancer to the kidney is rare. However, it is more frequently identified post-mortem, likely due to the asymptomatic nature and insufficient recognition.

**Figure 2:**
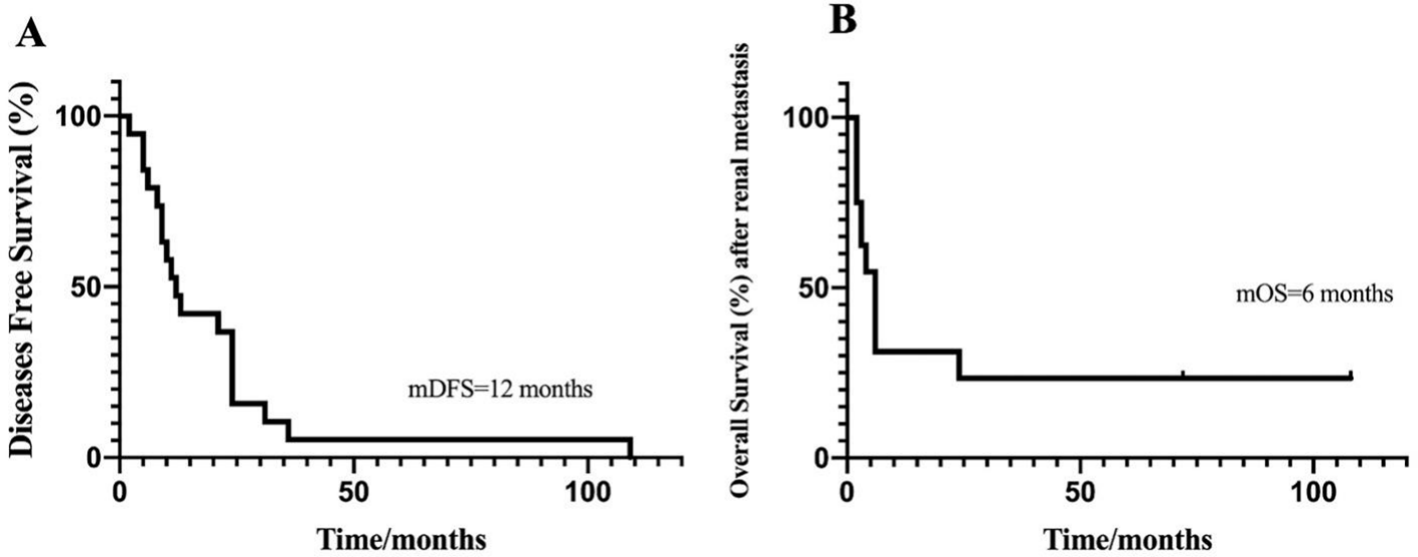
Kaplan-Meier analyses of prognosis according to the data from 21 cases: **(A)** Disease free survival curve (DFS), median DFS = 12 months. **(B)** Overall survival (OS) after renal metastasis, median OS = 6 months.

Until now, only 20 cases of esophageal cancer metastasis to the kidney have been reported (4, 5, 7–20) (**Figure 2**; **Table 1**). Analysis of these cases revealed the following characteristics: the age at onset ranged from 46 to 74 years, with an average of 54.6 years, and included 19 males and 1 female. The clinical manifestations were primarily back pain or hematuria, and some patients were asymptomatic. Most renal metastases were solitary. The onset of renal metastasis occurred between 2 and 36 months postoperatively. The preferred treatment regimens include radical nephrectomy with or without chemotherapy/radiotherapy, with survival post-metastasis ranging from 2 to 109 months. Our findings indicate that regular postoperative screenings among esophageal cancer patients can facilitate the timely detection of renal metastasis. The present case displays similar characteristics to those reported in the previous literature: specifically, a 71-year-old male patient who developed left kidney metastasis 109 months following surgery. For unilateral renal metastasis secondary to esophageal carcinoma, radical nephrectomy combined with platinum-based chemotherapy is the preferred treatment, which could effectively control local lesions and improve the patient’s prognosis. The reason is that surgical resection can lead to long-term disease-free survival and even potential cure, whereas radiotherapy is a local control modality that is unlikely to achieve radical eradication of established solid metastatic lesions. Although the kidney is radiosensitive, radiotherapy causes significant damage to the renal parenchyma and cannot easily deliver a curative dose. Furthermore, although ACC is sensitive to radiotherapy, the SCC component in mixed tumors is less radiosensitive and prone to developing radioresistance. In addition, surgery can provide a complete pathological assessment to guide subsequent treatment, and in selected cases, it allows simultaneous management of both the primary tumor and the metastatic lesion. The patient opted for partial nephrectomy, partly to prepare for subsequent chemotherapy and partly to improve the quality of life. Notably, the pathological type in this case is unusual, consisting of a rare combination of ESCC and adenoid cystic carcinoma, this mixed tumor presents unique characteristics. For instance, the patient in question still developed renal metastases despite negative postoperative lymph node status and clear surgical margins. The core causes lie in the preoperative occult micrometastases, distinct biological behaviors, and synergistic invasive properties of the two tumor components. First, negative lymph node status and clear surgical margins only indicate the absence of gross or microscopic tumor cells in the resected area and the submitted lymph nodes, but cannot rule out the presence of preoperative micrometastases. The esophageal squamous cell carcinoma (ESCC) component itself has a strong tendency for hematogenous metastasis. A core biological feature of adenoid cystic carcinoma (ACC) is its high local invasiveness and elevated long-term metastasis rate. Additionally, the number of lymph nodes submitted for pathological examination during surgery is limited (usually 20–30 nodes). Occult micrometastases may exist in unsubmitted lymph nodes, or tumor cells may only invade the extranodal capsule without forming microscopically detectable metastatic foci, leading to a false-negative pathological report. Second, ESCC combined with ACC is not a simple “coexistence” of two tumors, but rather involves synergistic biological behaviors that further increase the risk of distant metastasis. Third, the kidneys, as core organs of the urinary system, possess unique blood flow and tissue microenvironments that make them susceptible to becoming targets for tumor cell colonization. Fourth, the proliferation of residual minimal lesions is a secondary cause, which includes undetectable subclinical lesions that cannot be completely removed by surgery and the limitations of postoperative adjuvant therapy.

**Table 1:** Clinical pathological characteristics, treatments of the cases of kidney metastasis from esophageal cancer in the literatures.

Case	Age(years)	Gender	Pathology	Symptoms	The site of renal metastasis	Other metastasis site	Treatment	Time of metastasis to renal	Time of overall survival after renal metastasis/months
case1	61	male	squamous cell carcinoma	back pain and fever	left renal	none	nephrectomy+chemotherapy	11	2
case2	62	male	squamous cell carcinoma	not mentioned	not mentioned	none	not mentioned	not mentioned	2
case3	56	male	squamous cell carcinoma	hematuresis	single renal	none	nephrectomy	24	6
case4	62	male	squamous cell carcinoma	hematuresis	single renal	none	nephrectomy	5	6
case5	46	female	squamous cell carcinoma	back pain	right renal	none	nephrectomy+chemotherapy	8	6
case6	50	male	squamous cell carcinoma	back pain and hematuresis	right renal	none	nephrectomy+radiotherapy	24	4
case7	62	male	squamous cell carcinoma	no symptoms	left renal	none	nephrectomy+radiotherapy+chemotherapy	5	not mentioned
case8	57	male	squamous cell carcinoma	back pain	right renal	none	nephrectomy	2	longer than 3 months
case9	57	male	squamous cell carcinoma	back pain and hematuresis	right renal	none	nephrectomy	12	2
case10	74	male	squamous cell carcinoma	no symptoms	right renal	none	nephrectomy	13	3
case11	not mentioned	male	squamous cell carcinoma	no symptoms	single renal	none	nephrectomy	0	not mentioned
case12	64	male	squamous cell carcinoma	not mentioned	single renal	brain	not mentioned	36	longer than 9 years
case13	64	male	squamous cell carcinoma	no symptoms	left renal	none	nephrectomy+chemotherapy	24	not mentioned
case14	65	male	squamous cell carcinoma coexisting with lung adenocarcinoma	no symptoms	single renal	none	not mentioned	21	longer than 6 months
case15	46	male	squamous cell carcinoma	not mentioned	not mentioned	none	not mentioned	24	24
case16	63	male	squamous cell carcinoma	back pain	right renal	none	nephrectomy	9	3
case17	53	male	squamous cell carcinoma	back pain and hematuresis	left renal	none	nephrectomy	31	2
case18	46	male	squamous cell carcinoma	back pain	right renal	none	not mentioned	10	not mentioned
case19	47	male	squamous cell carcinoma	no symptoms	bilateral renal	none	not mentioned	9	not mentioned
case20	57	male	squamous cell carcinoma	back pain	bilateral renal	retroperitoneal lymph nodes	nephrectomy+radiotherapy	6	longer than 3 months
our case	71	male	squamous cell carcinoma coexisting with adenoid cystic carcinoma	no symptoms	left renal	none	nephrectomy+chemotherapy	109	longer than 9 years

In summary, negative postoperative lymph node status and clear surgical margins only signify the complete resection of local tumors, but cannot counteract the preoperative occult micrometastases of the mixed tumor or the characteristic long-term latent metastatic potential of the ACC component. This is precisely why long-term postoperative follow-up (including regular renal imaging examinations) is required for such patients, and personalized adjuvant treatment regimens should be formulated based on the molecular characteristics of the tumor components to reduce the risk of distant metastasis.

Squamous cell carcinomas primarily occur in the upper two-thirds of the esophagus, while adenocarcinomas are most commonly found in the lower one-third. The clinical manifestations of renal metastases closely resemble those of primary renal carcinomas, including hematuria, abdominal pain, and fever, sometimes these symptoms may be absent. The most common tumors that metastasize to the kidneys are melanoma, lung cancer, breast cancer, gastric cancer, gynecological tumors, and pancreatic cancer. Renal biopsy can be used to confirm the diagnosis of renal metastases.

For esophageal cancer with solitary renal metastasis, treatment options include radical nephrectomy or partial nephrectomy, followed by chemotherapy or radiotherapy. Among the 13 cases that underwent radical nephrectomy, survival time varied widely, ranging from 2 to 31 months with postoperative chemotherapy or radiotherapy. Currently, there are limited case reports on esophageal cancer metastasis to the kidney, most of which were solitary, and only four cases received postoperative adjuvant chemotherapy. Consequently, the efficacy of surgical and postoperative adjuvant treatments remains undetermined. In this case report, the patient underwent partial nephrectomy and received one cycle of first-line paclitaxel and platinum chemotherapy. The long-term therapeutic efficacy has yet to be evaluated.

Therefore, patients who have undergone radical resection for esophageal cancer should undergo regular and scheduled follow-up examinations as early as possible. Although renal metastasis from esophageal cancer is clinically rare, if a renal space-occupying lesion is detected, the possibility of secondary malignant renal tumors should be considered. Imaging examinations have limited value in differentiating the origin of the tumor, and histopathological examination is the gold standard for a definitive diagnosis. However, imaging examinations also constitute a core component of follow-up for patients with esophageal squamous cell carcinoma complicated with adenoid cystic carcinoma (ESCC+ACC), and play an irreplaceable role in the early detection of metastatic lesions, monitoring of local recurrence, and evaluation of treatment efficacy. Follow-up for this mixed tumor needs to balance early monitoring of hematogenous metastasis from the ESCC component and long-term screening for latent distant metastasis from the ACC component. It should focus on high-risk metastatic sites such as the kidneys, lungs and liver, while also paying attention to the risk of local recurrence. The risk of metastasis for this tumor persists throughout the long-term survival period. The characteristic of late recurrence of the ACC component dictates that follow-up must be continued for more than 10 years; screening should not be terminated simply because no recurrence is observed within 5 years after surgery. During the follow-up period, if symptoms such as lumbodorsal pain, hematuria, unexplained weight loss or fever occur, patients should undergo chest contrast-enhanced CT, renal contrast-enhanced MRI, serum tumor markers, and whole-body PET-CT as soon as possible. Early detection and confirmation of metastatic lesions can help formulate an effective and individualized treatment plan for the patient, thereby improving the prognosis.

Esophageal primary squamous cell carcinoma with an adenoid cystic carcinoma component is relatively rare, It has its own unique characteristics. ESCC is characterized by its aggressiveness and tendency for lymph node metastasis, whereas ACC is locally destructive but rarely metastasizes and shows a propensity for perineural spread.

In terms of treatment, surgery combined with chemotherapy remains the preferred approach. If the pathology indicates a relatively large proportion of the ACC component, postoperative radiotherapy is more effective than chemotherapy in controlling local disease, as ACC is relatively sensitive to radiation. If the pathology indicates a predominance of the ESCC component, postoperative chemotherapy is more appropriate, but the overall effect may be limited. In conclusion, the treatment of patients with esophageal primary squamous cell carcinoma accompanied by adenoid cystic carcinoma should be analyzed on an individual basis according to the specific circumstances.

## Data Availability

The original contributions presented in the study are included in the article/supplementary material. Further inquiries can be directed to the corresponding author/s.
